# Nanoscratching technique for highly oriented liquid crystal materials

**DOI:** 10.1038/s41598-018-27887-z

**Published:** 2018-06-21

**Authors:** Ahram Suh, Dong Ki Yoon

**Affiliations:** 1 Graduate School of Nanoscience and Technology, KAIST, Daejeon 34141 Republic of Korea; 2 Department of Chemistry and KINC, KAIST, Daejeon 34141 Republic of Korea

## Abstract

A simple, fast, and cost-effective technique to obtain highly oriented thermotropic and lyotropic liquid crystal (LC) phases using a simple nanoscratching method is presented. Highly aligned linear nanogrooves are fabricated by scratching substrates such as normal, indium tin oxide (ITO), curved glasses, and ITO-coated polyethylene terephthalate (PET) film using diamond lapping films. To demonstrate the feasibility of the platform, typical thermotropic and lyotropic LC materials in the nematic phase are used to demonstrate the well-aligned domains along with the resulting scratched nanogrooves. The polarised optical microscopy (POM) images show excellent dark and bright states depending on the sample rotation, proving that the LC molecules are well aligned. The electro-optical performance of the twisted nematic (TN) mode LC display fabricated using the nanogrooves is also measured and indicates reliable results compared with that of the conventional device. Indeed, scratch-induced nanogrooves are well generated on the curved substrate and ITO-coated PET film to show versatility of our technique. Our platform can suggest a new nanofabrication way to make various electro-optical devices as well as other patterning applications.

## Introduction

Nanofabrication techniques have been important for the current electronics. Among various kinds of fabrication tools, photolithography combined with etching process has been most widely used, though this is expensive and needs many additional processes. Recently, needs for nanofabrication has increased not only in electronic devices but also in many other applications such as biosensor, photonic crystal, and energy storage^1–6^. Thus, the cheap way to make nanopatterns is demanded, and bottom approaches using block copolymer, colloidal particles, and liquid crystals (LCs) have been developed to substitute photolithography though still there are some obstacles like prerequisites to use in the practical applications^7–17^. To solve this problem, a scratching method was proposed to make nanopatterns using an atomic force microscope (AFM) tip for scratching the given substrate, which forms various kinds of nanogrooves by controlling the load force between a contacted substrate and the AFM tip^18,19^. Inspired by this method, the simple scratching method using nano- or micron-sized diamond particle was developed to make large area-nanogrooves, which is very similar with sand paper^20–23^.

In this work, we report a simple nanoscratching technique for obtaining the highly oriented universal LC materials on nanogrooves. A highly aligned array of linear nanogrooves is effectively generated on normal, indium tin oxide (ITO), curved glass substrates, and ITO-coated PET film by scratching the materials with diamond lapping films, which induces planar alignment in both of thermotropic and lyotropic nematic LC phases. Here, thermotropic LC means the LC phases are transit by varying the temperature, while the phases of lyotropic LC are changed depending on concentration. The relationship between the diamond particle size and aligning efficiency is investigated by analysing topographic features with atomic force microscopy (AFM) as well as polarised optical microscopy (POM). The POM images and their angle-dependent polar plots show well-aligned LC phases in both cases. A twisted nematic (TN) mode LC display is fabricated using the array of linear nanogrooves, exhibiting reliable electro-optical properties as well as a reasonable response time. Also LC molecules are well aligned on a scratched curved substrate. Our simple method suggests a new means of aligning thermotropic and lyotropic LC structures within ~5 min, which is also very cost effective.

## Results and Discussion

**Figure 1:**
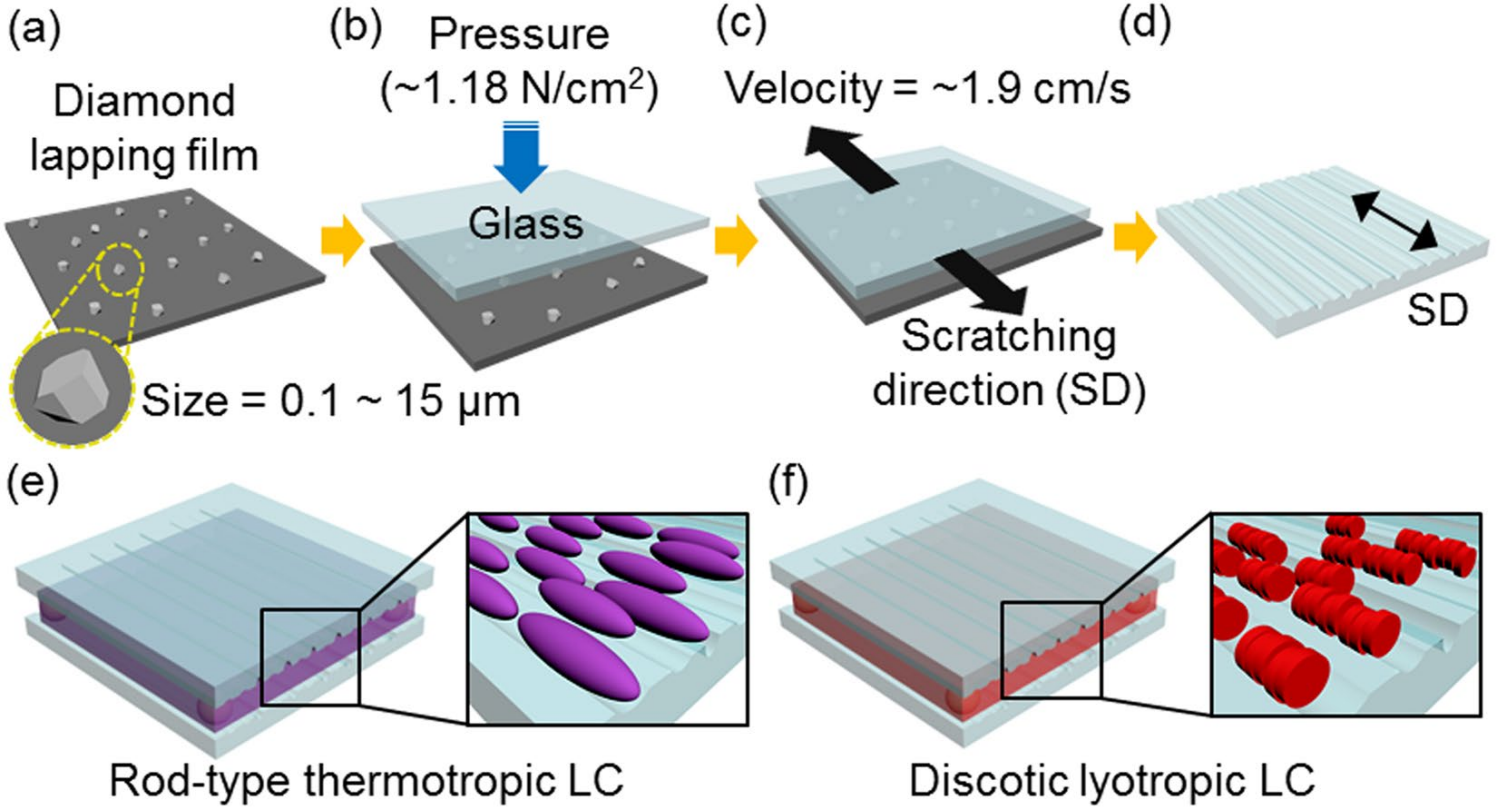
Experimental scheme to fabricate LC cells using a scratching method. (**a**) Embedded diamond particles on a polymer film. (**b**,**c**) Scratching process on a glass substrate. The black arrows indicate the scratching direction (SD). (**d**) Scratched glass substrate. The black double arrow indicates the SD. (**e**,**f**) Aligned rod-type thermotropic and discotic lyotropic LCs along the SD in the cell, respectively.

For the alignment of universal LC materials, common glass substrates (10 × 15 mm^2^) are prepared to create an array of nanogrooves by scratching the surface with diamond lapping films (Fig. 1). The size of the diamond particles embedded on the films is varied from 0.1 to 15 μm in diameter to determine the appropriate size for obtaining transparency and homogeneous alignment of LCs (Fig. 1a). To fabricate nanogrooves, the diamond lapping film is fixed on the bottom and the glass substrates are scratched forward and backward, with a constant pressure, ~1.18 N/cm^2^, and speed, ~1.9 cm/s, applied during the scratching process (Fig. 1b,c). Substrates are scratched back and forth 10 times which is enough to make the nanogrooves. The resulting scratched glass has the array of linear nanogrooves along the scratching direction (SD) (Fig. 1d). All scratching processes are repeated twice to create a sandwich cell, and the direction of the nanogrooves is matched to arrange LC molecules in a unidirectional alignment. The cell has a regular thickness, accomplished by placing 10 μm silica beads between two scratched substrates. Then the thermotropic and lyotropic LC materials are injected into the sandwich cell by a capillary process at the isotropic state and the nematic LC phase, respectively. The desired LC arrangement is shown in Fig. 1e,f, in which rod-type thermotropic LC molecules are aligned parallel to the SD, as well as the long axis of the columns made of discotic lyotropic LC molecules.

**Figure 2:**
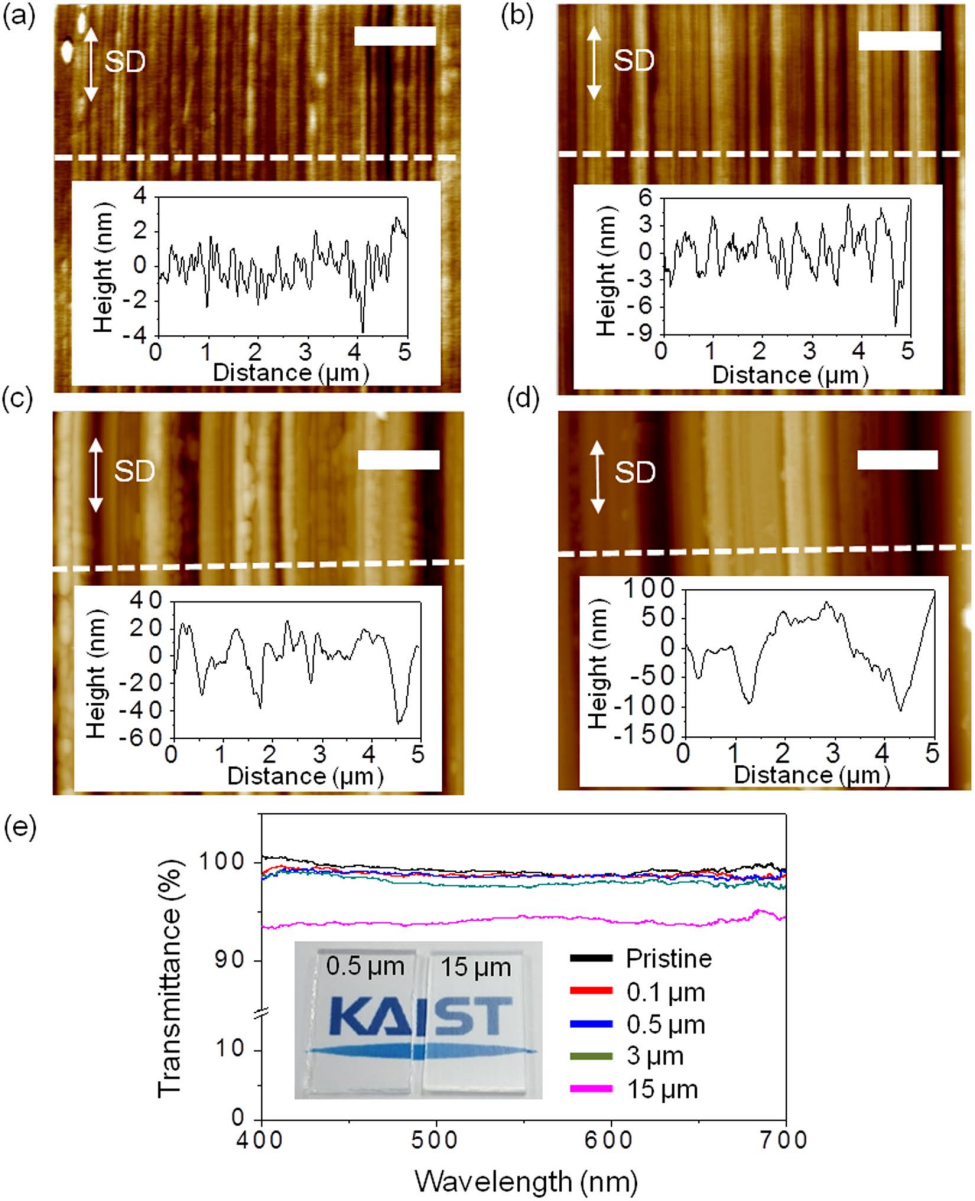
Characterization of scratch-induced nanogrooves on glass substrates. AFM images of glass substrates scratched with (**a**) 0.1 μm, (**b**) 0.5 μm, (**c**) 3 μm, and (**d**) 15 μm diamond particles in size. The white double arrows indicate the SD. All scale bars are 1 μm. Insets are height profiles along the white dotted lines in (**a**–**d**). (**e**) Comparison of transparency before and after the scratching process on the glass substrates with various sizes of diamond particles. Insets are real images of the scratched glass substrates with 0.5 μm (left) and 15 μm (right) sized diamond particles, respectively.

**Figure 3:**
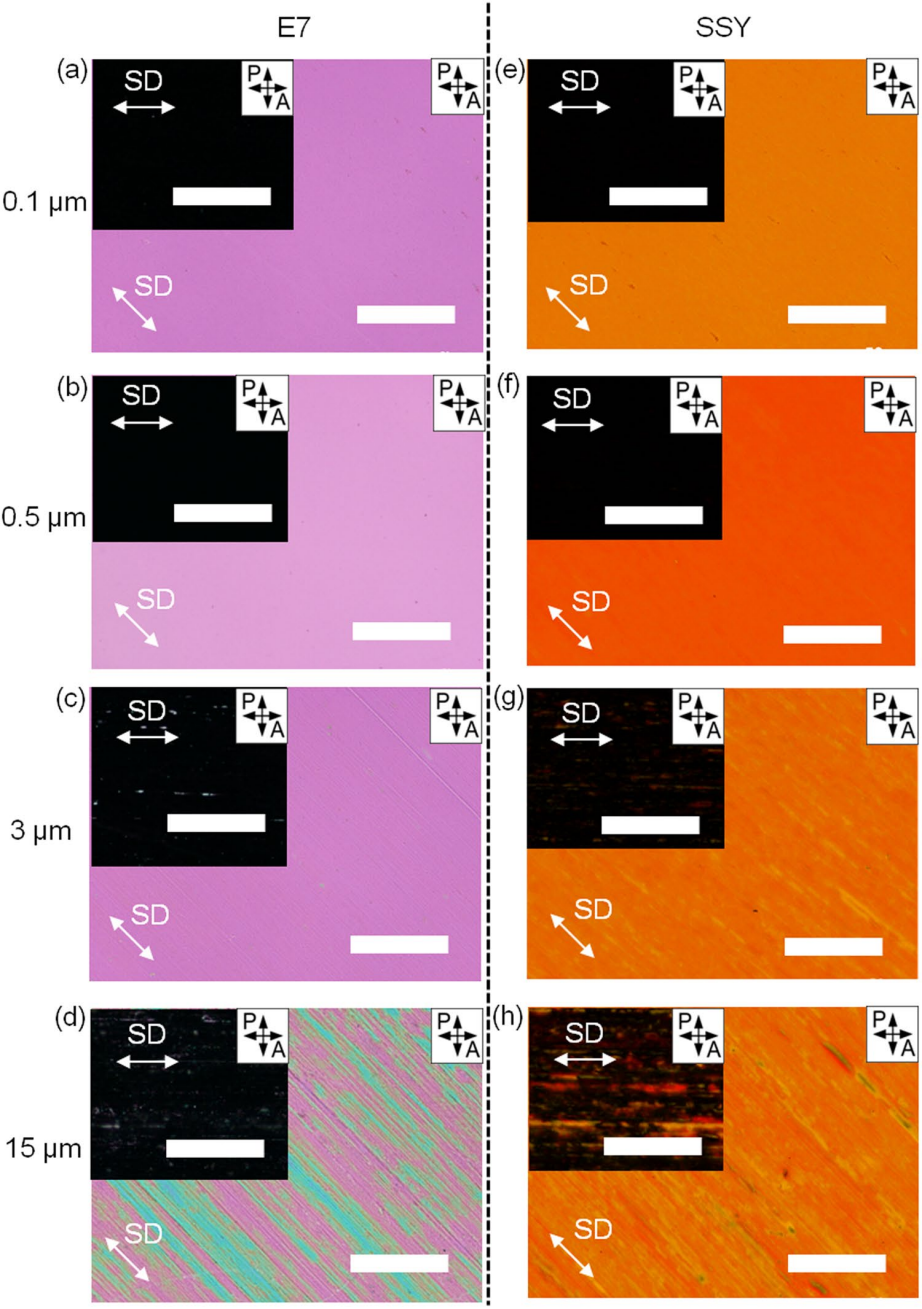
POM images of the alignment of E7 and SSY in nematic phase obtained by varying the size of diamond particles. The size of diamond particle is (**a**,**e**) 0.1 μm, (**b**,**f**) 0.5 μm, (**c**,**g**) 3 μm, and (**d**,**h**) 15 μm. Insets are POM images when the SD is parallel to the polariser axis. The white double arrows indicate SD. All scale bars are 100 μm.

The relationship between the diamond particle size and aligning efficiency is investigated by observing topographic features as well as the POM images (Figs 2 and 3). For this, the diamond particle sizes are varied to be 0.1 μm, 0.5 μm, 3 μm, and 15 μm. Direct observation of the scratched substrate is performed using atomic force microscopy (AFM), revealing the linear nanogrooves (Fig. 2). In the insets of Fig. 2a–d, the corresponding surface profile along the white dotted line is also measured to determine the roughness and depth, depending on the diamond particle size. The AFM images of glass substrates scratched with 0.1 μm and 0.5 μm-diamond lapping films show relatively smooth and uniform linear nanogrooves, revealing a low root-mean-square surface roughness (R_q_) of 1.09 nm and 2.23 nm, respectively. However, the substrates scratched with larger (3 μm and 15 μm) diamond particles show relatively irregular nanogrooves that exhibit large distributions in both depth and width (Fig. 2c,d). Their R_q_s are also very large: ~15.9 and ~46.6 nm, respectively. For the alignment application, transparency is one of the important factors, which is closely related with the roughness and can be also varied with the diamond particle size (Fig. 2e). Except for the case of the 15 μm-particle lapping film, the transmittance is ~ 100%, which is quite promising for real applications.

To observe the surface roughness-dependent alignment tendency of LC molecules (Fig. 3), two types of thermotropic and lyotropic LC molecules are prepared, which are E7 and 8CB (4′-n-octyl-4-cyano-biphenyl) for thermotropic LCs, and sunset yellow FCF (SSY) and disodium chromoglycate (DSCG) for lyotropic LCs. The LCs are injected into the cells by capillary force. In Fig. 3, the POM images for E7 and SSY results are shown. As expected from the AFM images (Fig. 2), homogeneous optical birefringence is observed in the thermotropic and lyotropic LC samples in the cells prepared with 0.1 μm and 0.5 μm-diamond lapping films, exhibiting no specific optical defects (Fig. 3a,b,e,f). The samples also exhibit completely dark images when the polarisers and SD are parallel and exhibit completely bright images at 45°. In this case, nanogrooves are regular with a finite periodicity as well as depth, which induce a uniform alignment of the nematic LC phase because the thermotropic and lyotropic LC molecules are well confined without any deformation. Based on these results, when compared to pristine and scratched cells using E7, 8CB, SSY, and DSCG, the impressive changes from typical schlieren textures to uniform aligned domains are observed (Fig. S1 in Supplementary Information). Since the length of the E7 and 8CB molecules in the molecular director direction is about 2.5 nm and the width is ~0.3 nm, and the SSY and DSCG discs have the radius of ~1 nm and the length of ~0.3 nm in the column direction, of which dimensions are much smaller than those of the nanogrooves, so the molecules and columns can be placed in the grooves. The LC cells made of nanogrooves scratched with the 3 μm-diamond lapping film reveal the less homogeneous optical textures with partially misaligned domains (Fig. 3c,g). However, dramatic changes in the optical textures are found in the cells prepared with the 15 μm-diamond lapping film, showing irregular domains (Fig. 3d,h).

**Figure 4:**
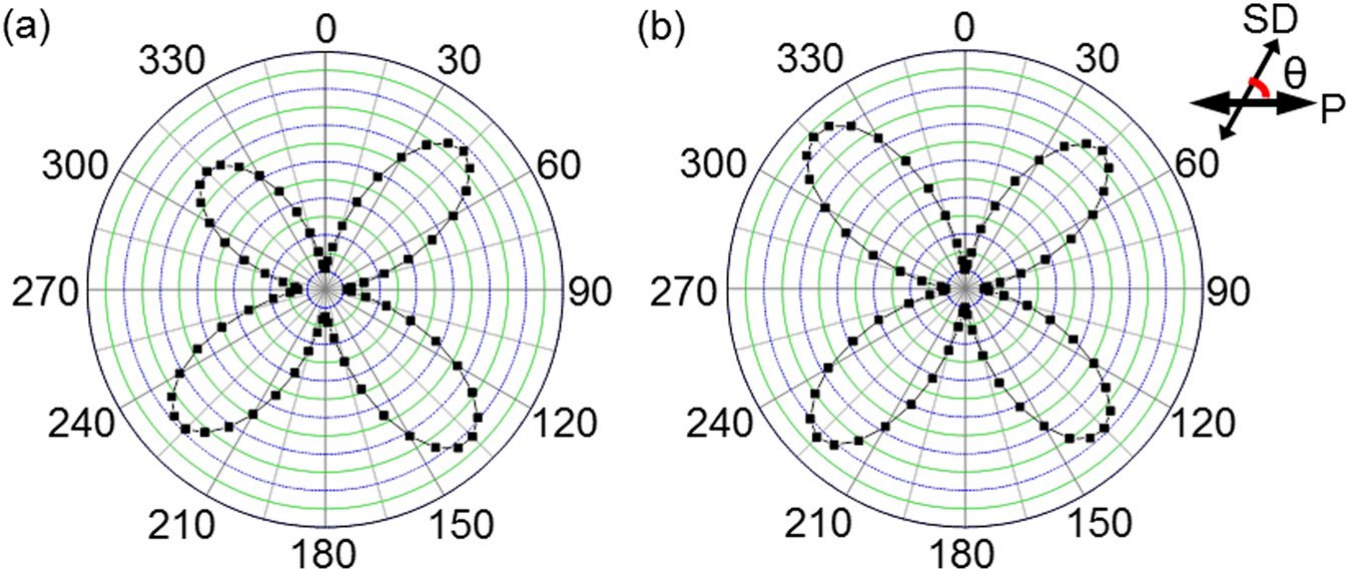
Polar plots of the uniaxial-oriented (**a**) E7 and (**b**) SSY cells. The polar plots show the transmittance change as the samples are rotated with respect to the polarisation of incident light.

To investigate the LC orientation of E7 and SSY, the polar plots are drawn in Fig. 4 to show 4-fold symmetry. The polar plots show the transmittance change as the samples are rotated with respect to the polarisation of incident light expected from POM images. In the polar plot, the highest transmittance is shown at θ = 45° + 90° × n, while the lowest transmittance is detected at θ = 90° × n (n is an integer). Here, θ is the azimuthal angle between the projection of the axis of rod-type molecules and columns onto the plane and the polariser. These results prove that both of rod-type molecules and columns of discotic molecules are highly oriented in one direction along the SD in equilibrium states without defects because their directors prefer to align along the groove direction to minimize the elastic distortion energy based on the elastic continuum theory of Berreman^24,25^.

**Figure 5:**
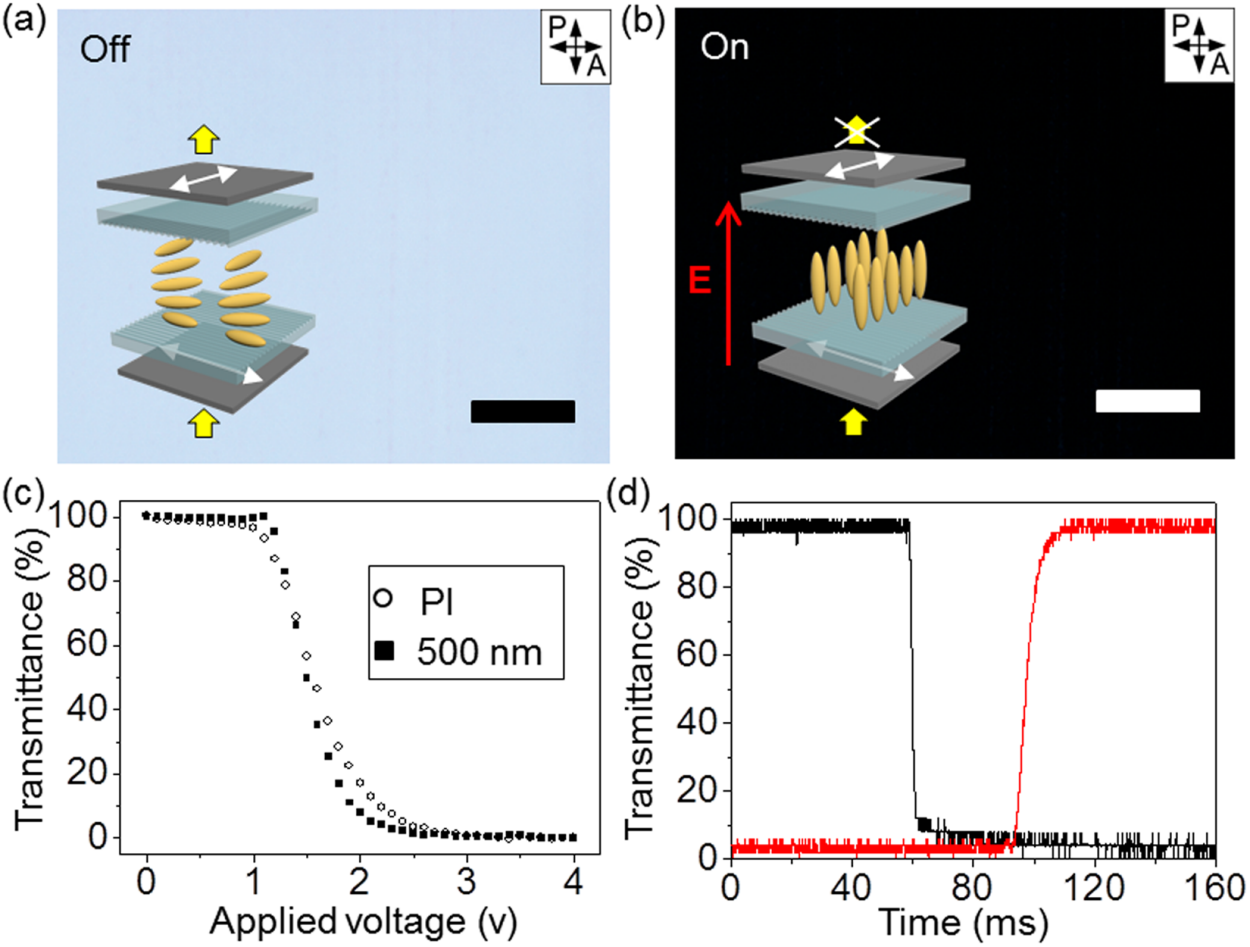
POM images of the TN mode LC display under crossed polarisers and the electro-optical properties of the fabricated displays. (**a**,**b**) POM images of the display with 5CB LC in the voltage (**a**) off state without an electric field and (**b**) on state with an electric field. Insets are schematic images of the TN mode LC display possessing nanogrooves. (**c**) V-T curves of the fabricated displays based on the scratching and polyimide (PI) rubbing methods. (**d**) Response time of the display based on scratching method. All scale bars are 100 μm.

To exploit our platform in display application, a TN mode LC display is fabricated (Fig. 5). A schematic of the TN mode LC cell with the scratched ITO glasses is shown in insets of Fig. 5a,b. Our device is normally in the ‘bright mode’, and thus the polarised light passing through the first polariser is guided by the nanogroove-mediated 5CB LC molecules when no electric field (**E**) is applied (inset of Fig. 5a). When the **E** is applied to the cell, most of the 5CB molecules become vertically aligned, parallel to **E**, which results in the dark mode, as shown in inset of Fig. 5b. As expected this scheme, 5CB molecules are gradually turned to the homeotropic alignment when the 4 V-**E** is applied to the cell, and thus a dark image is obtained (Fig. 5b). This simple example can prove our nano-scale grooves have low roughness and well guide LC molecules not to generate any defect structures. The corresponding electrical optic responses of the TN mode LCD are measured as a function of the applied voltage (Fig. 5c), in which the transmittance is varied from 100% to 0%. We obtain a smooth voltage-transmittance curve (closed square-line) without any optical bouncing, which is consistent with the POM images at the off- and on-states in Fig. 5a,b. As **E** increases, molecules are orient along to **E**, resulting in decreasing of transmittance. This gradual change in transmittance is important to gray scales of LC displays. A threshold voltage of the cell (V_th_) is approximately 1.2 V, which indicates the transmittance is gradually decreased in V-T curve when **E** exceeds the V_th_. And a driving voltage is approximately 1.9 V. The threshold and driving voltages are comparable with the conventional device made of the polyimide (PI) rubbing cell shown as open circle-line in Fig. 5c. The response time of LC molecules of our display is also measured (Fig. 5d), in which the rising and falling time are 3.55 and 9.07 ms, respectively. Total response times, which are sums of rising and falling times are 12.62 ms, which is comparable to the conventional LC device^26^. These results validate that our method can provide a simple and economic way to fabricate nano-patterns for display applications.

**Figure 6:**
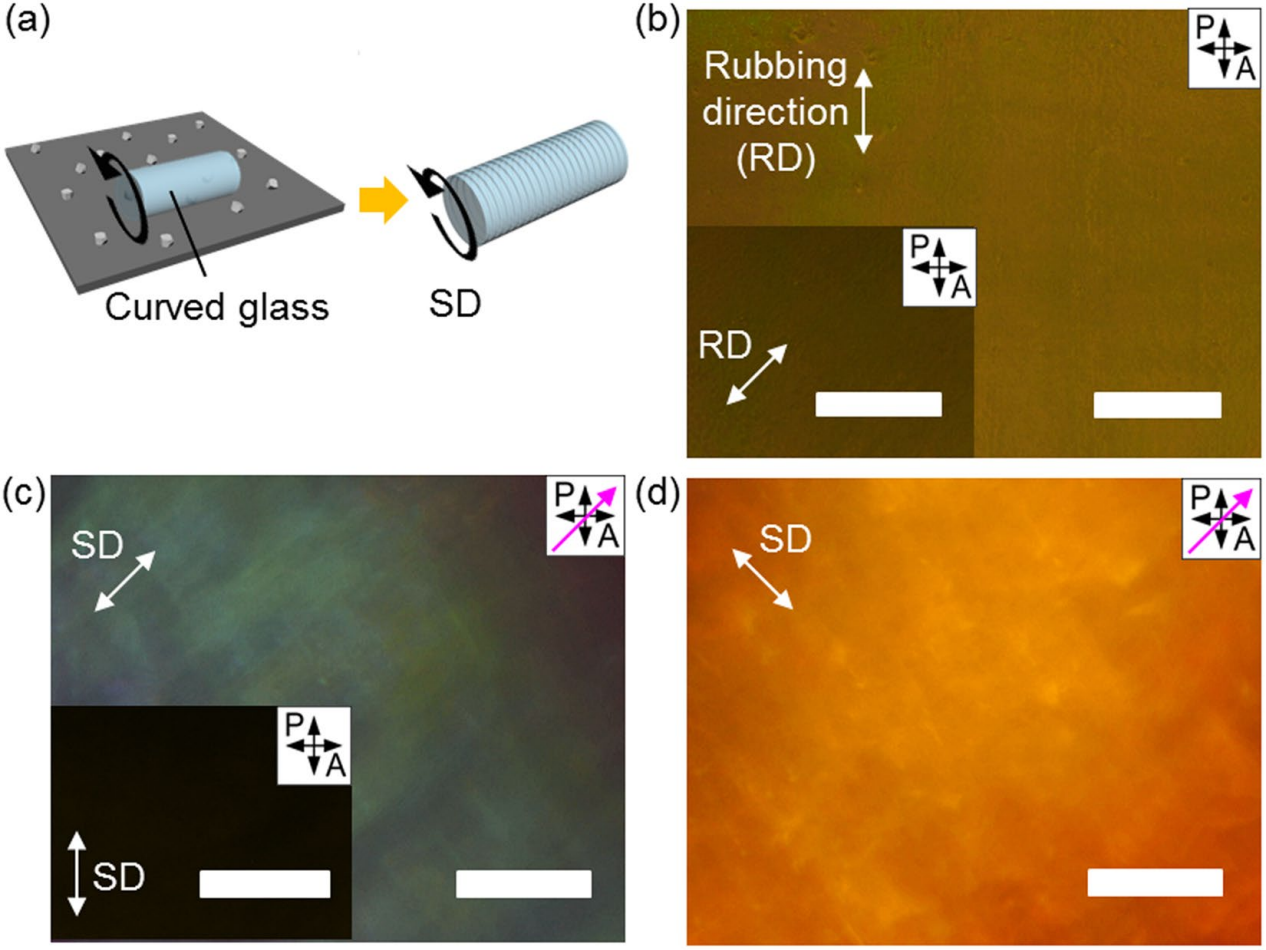
Alignment of 5CB on scratched curved glass substrate. (**a**) Experimental scheme to fabricate scratch-induced nanogrooves on curved glass. The black round arrow indicates the SD. (**b**) POM images of misaligned 5CB on the PI coated and rubbed surface. (**c**,**d**) POM images of well-aligned 5CB on scratched surface. All scale bars are 200 μm.

We also present the versatility of our simple scratching method by applying on the various substrates such as curved surface and ITO-coated PET film (Fig. 6 and Fig. S2 in Supplementary Information). To fabricate the scratched surface on a curved glass substrate, the glass is rotated on the 0.5 μm-diamond lapping film in one direction (Fig. 6a). The resulting scratched curved glass has the array of linear nanogrooves along the SD. In the case of a curved glass with conventional method based on rubbed PI layer, molecules are poorly aligned which does not show apparent difference in the brightness when the sample is rotated with respect to the polariser (Fig. 6b). This is because PI layer cannot be uniformly coated on the curved substrate. Compared with the rubbed PI sample, 5CB molecules are uniformly aligned along the SD on the scratched surface, showing a homogeneous optical birefringence (Fig. 6c,d). The sample also exhibits completely dark image when the polarisers and SD are parallel and exhibits a bright image at the 45° rotated state. To precisely determine the director of molecule, first-order retardation plate (λ = 530 nm) with a slow axis along the pink arrow is inserted. Cyan-blue and yellow colour images indicate that rod-type 5CB molecules are well oriented along with the SD and perpendicular to the SD, respectively. We also demonstrate that 5CB molecules are successfully aligned on the scratched ITO-coated PET film and fabricate the TN mode display with the scratched film, showing the reliable electro-optical result (Fig. S2 in Supplementary Information). The PET film is easily deteriorated by heat, so that the conventional PI coating and thermal annealing cannot be used. However, our method can be applied to this polymer film inducing highly oriented 5CB molecules on the scratched ITO-coated PET film. The POM images show the bright and dark states in the electric field-off and on-state, respectively, which proves that 5CB molecules are well aligned in the cell. These results demonstrate that our method is applicable to various substrates which are difficult to align LC materials using conventional methods.

To exploit our method in the further application, we investigate the alignment of gold nanorods which are considered to be the lyotropic LC at a certain concentration on the Si wafer scratched with 0.1 μm-diamond lapping film (Fig. S3 in Supplementary Information). Scanning electron microscopy (SEM) images clearly show that gold nanorods are linearly oriented along the nanogrooves in the single line marked in yellow, while gold nanorods are randomly assembled on the pristine Si wafer. This validates that our method is a promising tool to obtain highly ordered and oriented particles that are applicable to opto-electronic applications^27^.

## Conclusions

We have proposed a homogeneous planar alignment for thermotropic and lyotropic LC molecules by introducing scratch-induced nanogrooves via a simple, fast, and economic process. The nanogrooves are systematically generated depending on the diamond particle size, forcing rod-type thermotropic LC molecules and columns of discotic lyotropic LC molecules to be highly oriented along the SD. A TN mode LCD device with scratched ITO substrates is successfully fabricated, and its electro-optical properties are also comparable to conventional one. Furthermore, LC molecules are well aligned on the scratched curved substrate. Our results might contribute to the alignment of other soft materials on various substrates without the conventional polymer alignment layer, which can be effectively used in various electro-optical devices as well as in patterning applications.

## Methods

### Materials and Methods

Bare glass substrates (10 × 15 mm^2^) were chemically cleaned with acetone, ethanol, and deionized water to remove any organic residue. The glass substrates with nanogrooves were fabricated by scratching the surface with diamond lapping films (purchased from Allied High Tech Products., Inc.). The sizes of the diamond particles embedded on films were 0.1 μm, 0.5 μm, 3 μm, and 15 μm. The film was fixed on the bottom and the glass substrate was scratched forward and backward. The applied pressure on the glass substrate during scratching was ~1.18 N/cm^2^ and the scratching velocity was ~1.9 cm/s. Substrates were scratched total 10 times which is enough to make the nanogrooves. This scratching process induced nanogrooves on the substrate in one direction. After the scratching process, the substrate was chemically cleaned again with sonication to remove any particles. Two scratched glass substrates were sandwiched together in one direction with 10 μm silica beads. To fabricate the TN cell, two ITO glass substrates were sandwiched orthogonally. Electro-optical performance was measured using a 1 kHz electric field. To fabricate the scratched curved glass, the glass was rotated to scratch. For thermotropic LCs, E7, 8CB (4′-n-octyl-4-cyano-biphenyl), and 5CB (4-cyano-4′-pentylbiphenyl) were used (used as purchased from SYNTHON Chemicals GmbH & Co. and Sigma Aldrich). For lyotropic LCs, SSY and DSCG (used as purchased from Sigma Aldrich) were dissolved in deionized water at concentrations of 31 wt% and 16 wt%, respectively, and gold nanorods were synthesized by seed-mediated growth method. The LC materials were injected into the sandwich cell by a capillary manner.

### Characterization

The AFM images of the scratched substrates were obtained in contact mode (Bruker, Multimode-8). All polarised optical images were obtained using POM (LV100POL, Nikon). The transmittance of the scratched substrates and the TN cell was measured using a customized optical microscope equipped with a USB-2000+ spectrometer (Ocean Optics). The electric field was generated by a function generator (33210 A, Agilent). Assembled gold nanorods were directly observed using a field-emission scanning electron microscope (FE-SEM; S-4800, HITACHI) after a 2 nm-thick platinum coating.

## Electronic supplementary material

Supplementary Information: Nanoscratching technique for highly oriented liquid crystal materials
