# Supplementary material for: Nanoscratching technique for highly oriented liquid crystal materials
Source: Sci Rep. 2018 Jun 21;8:9460. doi: 10.1038/s41598-018-27887-z (PMC6013491; doi:10.1038/s41598-018-27887-z)
Supplement: Supplementary file 1 — Supplementary Information: Nanoscratching technique for highly oriented liquid crystal materials [file 41598_2018_27887_MOESM1_ESM.docx]

**Supplementary Information: Nanoscratching technique for highly oriented liquid crystal materials**

Ahram Suh1, Dong Ki Yoon1,2,*

1Graduate School of Nanoscience and Technology, KAIST, Daejeon, 34141, Republic of Korea

2Department of Chemistry and KINC, KAIST, Daejeon, 34141, Republic of Korea

*nandk@kaist.ac.kr


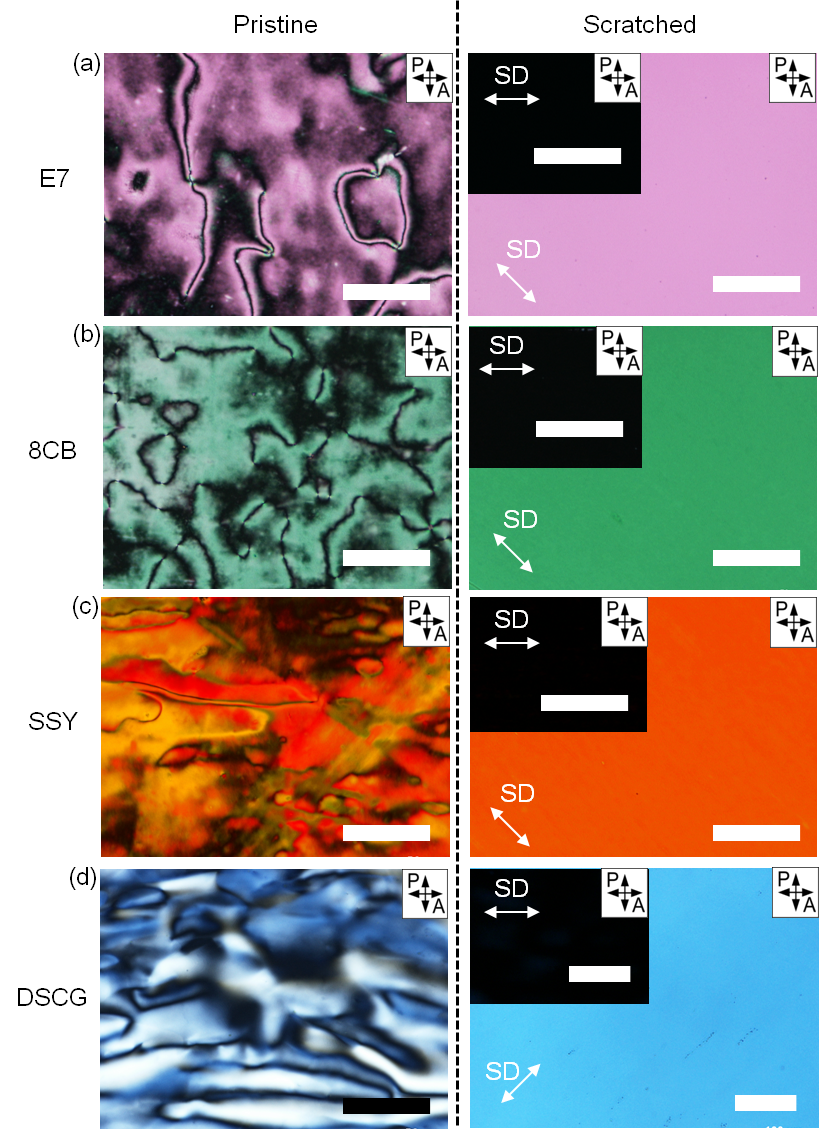


**Figure S1.** Alignment behaviours of the nematic phase of E7, 8CB, SSY, and DSCG in pristine cells and scratched cells prepared with 0.5 μm-diamond lapping film. The POM images of random schlieren and homogeneous planar texture of (a) E7, (b) 8CB, (c) SSY, and (d) DSCG in pristine and scratched cells, respectively. Insets show the POM images when the SD is parallel to the polariser axis. The SD is indicated by the white double arrows. All scale bars are 100 μm.


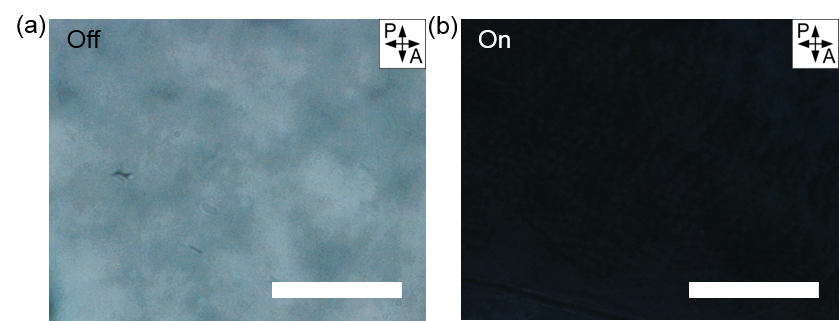


**Figure S2.** POM images of the TN mode LC display prepared with ITO-coated PET film under crossed polarisers. (a, b) POM images of the display with 5CB LC in the voltage (a) off state without an electric field and (b) on state with an applied electric field. All scale bars are 50 μm.


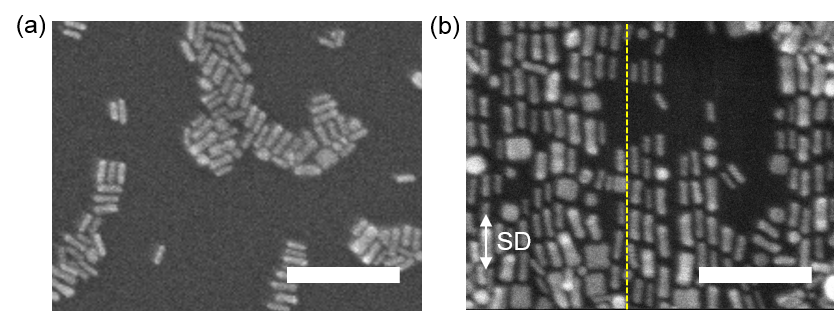


**Figure S3.** SEM images of assembled gold nanorods using dip-coating method on the pristine and scratched Si wafers. (a) Randomly assembled gold nanorods are found on the pristine Si wafer. (b) Linearly aligned gold nanorods on the scratched Si wafer prepared with 0.1 μm-diamond lapping film are shown. The SD is indicated by the white double arrows. Gold nanorods are oriented in the single line marked in yellow dashed line. All scale bars are 200 nm.
